# Efficient N-tailing of blunt DNA ends by Moloney murine leukemia virus reverse transcriptase

**DOI:** 10.1038/srep41769

**Published:** 2017-02-02

**Authors:** Yoshiyuki Ohtsubo, Yuji Nagata, Masataka Tsuda

**Affiliations:** 1 Department of Environmental Life Sciences, Graduate School of Life Sciences, Tohoku University, 2-1-1 Katahira, Sendai 980-8577, Japan

## Abstract

Moloney murine leukemia virus reverse transcriptase (MMLV-RT) is a widely used enzyme for cDNA synthesis. Here we show that MMLV-RT has a strong template-independent polymerase activity using blunt DNA ends as substrate that generates 3′ overhangs of A, C, G, or T. Nucleotides were appended efficiently in the order A > G > T > C, and tail lengths varied from 4 to 5, 2 to 7, 2 to 4, and 2 to 3 for A, C, G, and T, respectively. The activity was so strong that nearly all our test DNA ends were appended with at least one A, C, G, or T. The N-tailing activity of MMLV-RT was enhanced in the presence of Mn^2+^, and the G-, C-, and T-tailing activities were further enhanced by dCMP, dGMP, and dAMP, respectively. This is the first report of an enzymatic activity that almost thoroughly appends two or more As, or one or more Cs, Gs, or Ts to the 3′ end of double-stranded DNA, which would enable exhaustive analysis of DNA samples. The N-tailing activity of MMLV-RT is potentially useful in many biotechnological applications.

Ligation of two DNA molecules with one containing a single 3′ A-overhang and the other a single 3′ T-overhang is universally used in a wide range of applications, including TA-cloning^1^,^2^, GC-cloning^3^, and library preparations for next-generation sequencing^4^,^5^. Single 3′ A-overhangs are added using the template-independent activity of DNA polymerases such as Taq DNA polymerase from *Thermus aquaticus*^6^, exo^−^ mutant of the Klenow fragment of *Escherichia coli* DNA polymerase I^7^, DNA polymerase IV from *Sulfolobus solfataricus*^8^, DNA polymerase alpha from chick embryo^6^, rat DNA polymerase beta, DNA polymerase I from *Saccharomyces cerevisiae*^6^, and reverse transcriptase (RT) from avian myeloblastosis virus^6^ and human immunodeficiency virus (HIV)^9^. These polymerases preferentially add a single A to the 3′ end of blunt-ended DNA, and addition of two or more nucleotides has not been observed except for DNA polymerase IV from *S. solfataricus*, which exhibits significantly less efficient incorporation of the second dATP^8^. RT from HIV extends as many as four adenines on a DNA-RNA template following complementary strand synthesis, but shows a limited activity on double-stranded DNA ends^9^. To date, no polymerase that efficiently appends two or more As, or one or more Cs, Gs, or Ts to the double-stranded DNA 3′ end has been identified.

Only a few techniques to create 3′ overhangs have been reported in addition to the use DNA polymerases. Single 3′ T-overhangs are generated using terminal deoxynucleotidyl transferase and dideoxythymidine triphosphate (ddTTP)^10^ or using synthetic oligonucleotides that anneal to create double-stranded DNA with a T-overhang at one 3′ end^4^,^5^.

Moloney murine leukemia virus reverse transcriptase (MMLV-RT)^11^, widely used for cDNA synthesis, also has DNA polymerase activity using DNA as a template. In fact, this enzymatic activity has been used in cDNA cloning^12^,^13^,^14^,^15^. When MMLV-RT arrives at the 5′ end of an RNA template, it preferentially adds Cs to the cDNA end. In the presence of a single-stranded DNA often called template-switching oligonucleotide (TSO), which contains a stretch of ribonucleotide Gs at its 3′ end, MMLV-RT switches template from RNA to TSO, and synthesizes DNA that is complementary to TSO and covalently bound to the cDNA. It also appends Cs to the newly synthesized DNA and undergoes template switching, thereby generating concatenated TSO sequences^14^. This activity suggests that MMLV-RT may also have the ability to add dCTP or other deoxynucleotides to blunt ends of double-stranded DNA.

In this study we tested the ability of MMLV-RT to add deoxynucleotides to the ends of DNA molecules. MMLV-RT shows such a strong activity to add a few As, Cs, Gs, and Ts to the 3′ end of blunt-ended DNA that most molecules are tailed at the end of the reaction. The high nucleotide addition efficiency and a putative high thermal stability of longer overhangs make MMLV-RT an attractive enzyme to be used in various additional biotechnological applications.

## Materials and Methods

### MMLV-RT assay

Wild-type MMLV-RT (200 U/μL) was purchased from Nippon Gene (Japan) and a variant MMLV-RT (100 U/μL) was purchased from Clontech Laboratories (US-DE). In our experiments, both enzymes exhibited essentially the same tailing activities. Another variant MMLV-RT (TOYOBO, Japan) also exhibited tailing activity, although we have not tested it in detail (data not shown). dATP, dCTP, dGTP, and dTTP solutions (100 mM) were purchased from TAKARA (Japan). Deoxynucleoside monophosphates (dAMP, dCMP, dGMP, and dTMP) were purchased from Sigma-Aldrich and dissolved in TE buffer (10 mM Tris-HCl and 1 mM EDTA) at a concentration of 100 mM. Reactions were carried out in PCR tubes using a thermal cycler (C1000; Bio-Rad) at 30 °C. The reaction mixture contained, in a total volume of 10 μL, 20 to 500 fmols of substrate DNA, 2 μL of 5 × 1st strand synthesis buffer for SMARTScribe Reverse Transcriptase (250 mM Tris-HCl pH 8.3, 375 mM KCl, and 30 mM MgCl_2_), 2 mM DTT, 4 mM dATP, dCTP, dGTP, or dTTP, 4 mM MnCl_2_, and 50 U MMLV-RT. Deoxynucleoside monophosphates were added at a final concentration of 10 mM only when explicitly stated. An aliquot of 0.5 μL of the reaction mixture was analyzed by a capillary sequencer (see below).

### DNA quantification

DNA labeled with the FAM fluorophore was quantified using an Infinite 200 fluorescence spectrophotometer (TECAN, Swiss). Primer SA560, which contains a FAM at its 5′ end, was used to make a calibration curve.

### Blunting of DNA ends

Blunting of DNA ends was conducted by treating DNA with KOD DNA polymerase (TOYOBO, JAPAN) at 60 °C for 10 min according to the manufacturer’s instructions.

### DNA preparation

All DNA molecules were obtained by PCR using plasmid pGiTp (a synthetic plasmid, see Supplementary Fig. S2) as template. A 300-bp DNA molecule was amplified using primers SA560 (with FAM at the 5′ end) and SA585, and the product was digested with PvuII. The resulting 70-bp blunt-end molecule with FAM at one 5′ end (FAM70) was purified by polyacrylamide gel electrophoresis. Nucleotide addition occurred at the 3′ end of the FAM-labeled strand.

Primer SA560 was used with primers SA651_A, SA649_C, SA648_G, and SA650_T to obtain products FAM69A, FAM69C, FAM69G, and FAM69T, respectively, which were purified as described above. DNA molecules with 140, 280, 560, 840, and 1,120 bp were amplified using primer SA560 with primers SA643, SA644, SA645, SA646, and SA647, respectively. These last five primers share the same seven nucleotides (CTGTCTC) at their 5′ ends. DNA molecules with 207, 560, 1,039, and 2,000 bp were amplified using primer SA645 with primers SA101, SA102, SA103, and SA104, respectively. Oligonucleotides were purchased from Eurofins Genomics (Tokyo, Japan) and are listed in Supplementary Table S1.

### DNA length analysis

Ten microliters of GeneScan –500 LIZ or GeneScan –1200 LIZ Size Standard (Thermo Fisher Scientific, US-MA) were added to 1 mL HiDi formamide (HD-LIZ). An aliquot of 0.5 μL of the reaction mixture was added to 12.5 μL of the HD-LIZ solution, vortexed, heated for 3 min at 96 °C, and then analyzed using a 3130xl Genetic Analyzer (Thermo Fisher Scientific, US-MA), a G5 dye set, the POP-7 polymer, and 50-cm capillaries. Sequencing data was analyzed using TraceViewer (http://www.ige.tohoku.ac.jp/joho/traceviewer/), and two LIZ bands were used to calibrate electrophoresis. To determine the G-tailing products of DNA molecules with 207, 560, 1,039, and 2,000 bp, the tailing reaction mixture was purified by phenol/chloroform/isoamylalcohol extraction and ethanol-precipitation, digested with DraII, and purified again. Double-stranded FAM-labeled DNA with an overhang complementary to DraII cleavage ends was obtained by annealing primers SA201 and SA202 and purifying using polyacrylamide gel electrophoresis. The resulting adapter molecule was ligated to DraII-digested products using a DNA ligation kit (TAKARA) and then analyzed by capillary sequencing as described above.

## Results

### Determination of optimal reaction conditions

**Figure 1:**
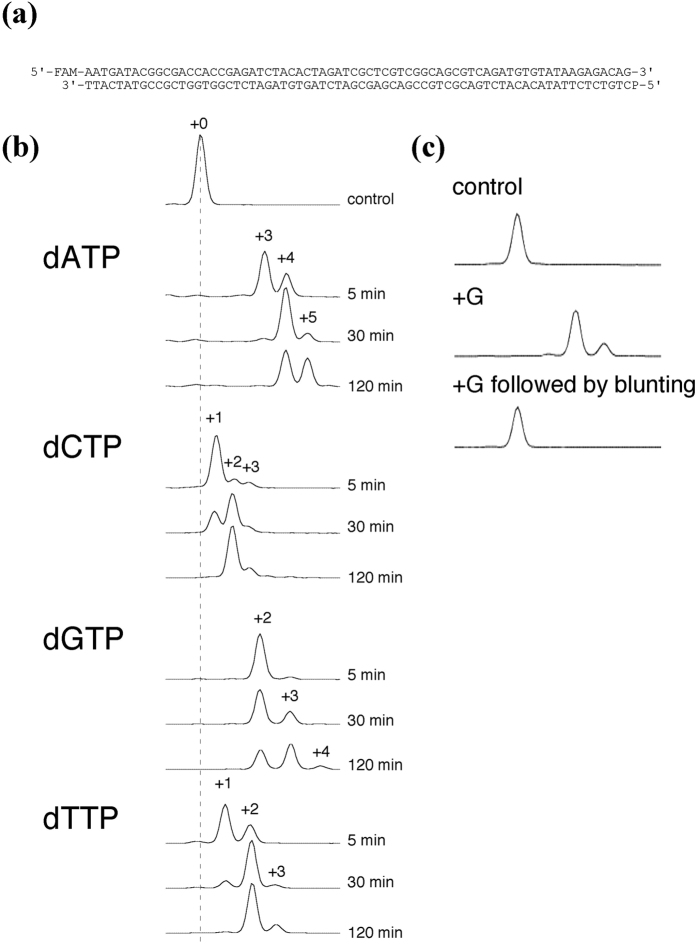
MMLV-RT shows high levels of tailing activity. (**a**) FAM70 DNA substrate. The 3′ end of the FAM-labeled strand is the tailing target. P, phosphate group. (**b**) MMLV-RT generated tails of A, C, G, and T. Ten nanomolar FAM70 DNA was incubated with MMLV-RT in the presence of the indicated deoxyribonucleoside triphosphates for 5, 30, and 120 min. Unreacted controls are shown at the top. For some peaks, relative sizes are indicated. (**c**) Tails were removed by blunting. Ten nanomolar FAM70 DNA was incubated with MMLV-RT in the presence of dGTP for 2 h, purified by phenol/chloroform/isoamylalcohol extraction and ethanol precipitation, and then subjected to blunting by KOD polymerase.

To explore the template-independent polymerase activity of MMLV-RT, FAM70 DNA (Fig. 1a) was used as a tailing substrate. FAM70 carries a 5′ phosphate group at the non-FAM-labeled end, the standard phosphorylated end of DNA molecules. The optimal reaction conditions for the tailing of FAM70 were first determined. Optimal concentrations of dATP, dCTP, dGTP, and dTTP were 4, 7, 4, and 6 mM, respectively, and the optimal Mn^2+^ concentrations were 2 mM for A-tailing and 4 mM for C-, G-, and T-tailing (data not shown). For convenience, reactions were performed in 4 mM dNTPs and 4 mM MnCl_2_. Several temperatures were also tested (27 °C, 30 °C, 37 °C, 40 °C, and 42 °C) for the G-tailing reaction (5 min incubation at 1 mM dGTP in the absence of MnCl_2_), and 40 °C was found to be optimal (data not shown). However, heating of MMLV-RT at 42 °C for 15 min resulted in loss of 50% of activity^16^, and our data showed that G-tailing activity at 30 °C corresponded to 93% of that at 40 °C. We therefore conducted the tailing experiments at 30 °C. Among the dCMP concentrations tested (1, 2, 5, 10, 15, 20, and 25 mM), G-tailing was most efficient at 10 or 15 mM (data not shown). No obvious increase in tailing activity was observed by changing the concentration of buffer (0.1 to 2×), or by adding PEG 6000 (1% to 10%), DMSO (2% to 20%), MgSO_4_ (0.5 to 5 mM), NaCl (10 to 100 mM), or BSA (0.002% to 0.02%).

### Highly efficient nucleotide addition by MMLV-RT

To evaluate the tailing efficiency of MMLV-RT, FAM70 DNA was incubated with the enzyme in the presence of dATP, dCTP, dGTP, or dTTP and analyzed under denaturing conditions in a capillary sequencer. As shown in Fig. 1b and Supplementary Fig. S1a, most, if not all, of the substrate DNA molecules acquired at least one nucleotide in all reactions. A-tailing reactions occurred markedly faster and with longer extension products (+A4 and +A5) compared to other reactions. In C-tailing reactions, a fraction of substrate molecules acquired C-tail extensions as long as +C7 (Fig. S1a). G-tailing reaction products were mostly +G2 and +G3, and formation of +G2 products was far faster than that of +G3 products. The major T-tailing reaction product was +T2, and +T4 products were rarely observed.

### Overhanging nucleotides in reaction products

To investigate whether addition of nucleotides generated 3′ overhangs, DNA molecules were incubated with MMLV-RT in the presence of dGTP, extracted with phenol/chloroform, precipitated with ethanol, and incubated with KOD polymerase to generate blunt ends (Fig. 1c). As expected, the size of the FAM-labeled strand reverted back to its original size, indicating that the MMLV-RT-treated DNA contains a 3′ overhang.

### Positive effects of dNMPs on tailing reactions

**Figure 2:**
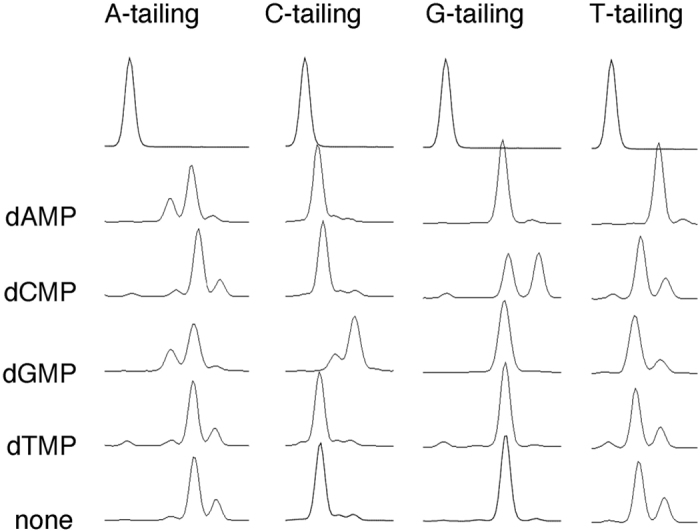
dGMP, dCMP, and dAMP specifically enhance C-tailing, G-tailing, and T-tailing, respectively. Ten nanomolar FAM70 DNA was subjected to A-, C-, G-, and T- tailing in the presence of 10 mM dAMP, dCMP, dGMP, or dTMP for 5 min. TE buffer was added as control. The unreacted control of each tailing reaction is shown at the top.

Previous work by Clark *et al*. showed that the +1 blunt-end incorporation of dNTP by the Klenow fragment is enhanced in the presence of complementary dNMPs^7^. These results suggest that dNMPs may enhance the tailing activity of MMLV-RT. To test this hypothesis, different dNMPs were added to the tailing reactions. As expected, of the sixteen possible combinations, dAMP, dCMP, and dGMP specifically enhanced T-, G-, and C-tailing, respectively (Fig. 2). The A-tailing reaction was not enhanced by the addition of the four dNMPs, and was adversely affected by the addition of dAMP and dGMP (Fig. 2).

### Effect of the 3′ terminal nucleotide on tailing reactions

**Figure 3:**
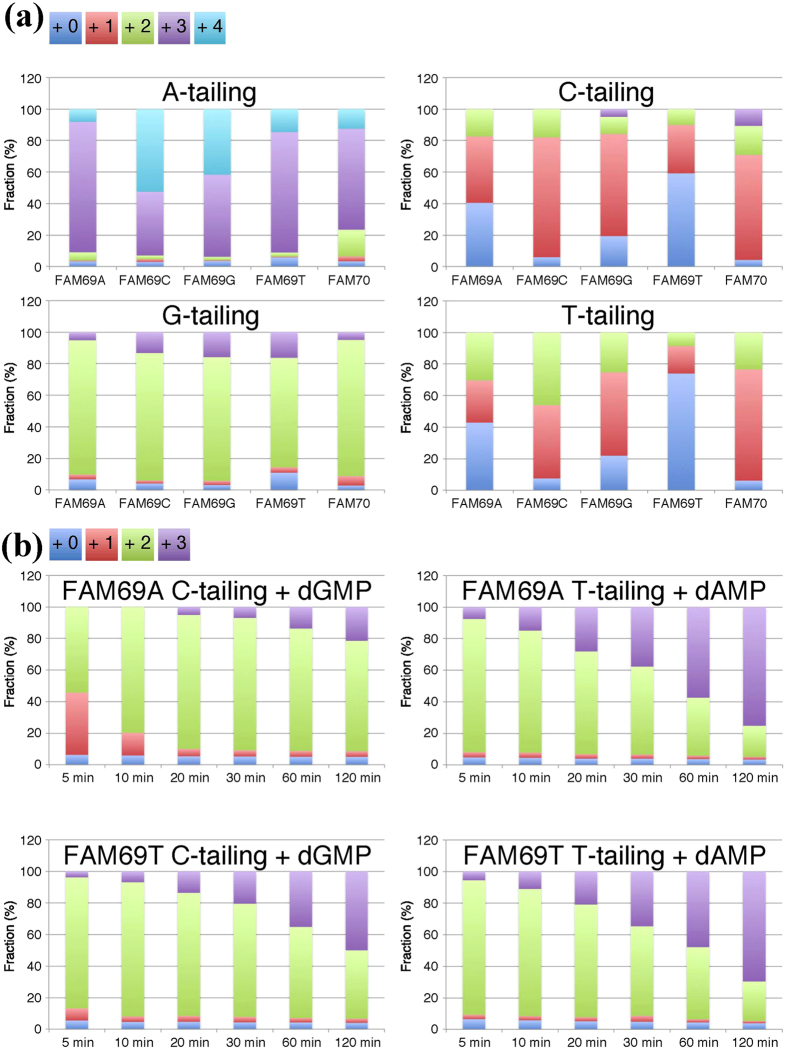
Less efficient C- and T-tailing by MMLV-RT of DNA substrates with A or T at the 3′ end that is alleviated by dGMP and dAMP. (**a**) Tailing efficiencies on different DNA ends. Five different DNA molecules at 10 nM were subjected to A-, C-, G- or T-tailing for five min, and the size distribution of the reaction products were analyzed. (**b**) dNMPs enhanced less efficient tailing. FAM69A and FAM69T DNAs were subjected to C- and T-tailing reactions in the presence of dGMP and dAMP, respectively. Results are shown as averages of triplicate experiments.

From a biotechnological point of view, it is important to characterize the MMLV-RT-mediated tailing reactions on DNA targets with different nucleotides at the 3′ terminus. To that end, four kinds of FAM-labeled 70-bp DNA molecules were prepared by PCR (all lacking a 5′ phosphate group). The nucleotide sequences of FAM69G and FAM70 were identical, and those of FAM69A, FAM69C, and FAM69T were identical to FAM70 except that the last base at the 3′ end of the FAM-labeled strand was A, C, or T, respectively. These five FAM-labeled DNAs were subjected to the tailing reactions, and enzymatic activity was interrupted after five min to determine the terminal-nucleotide preference (Fig. 3a). The speed of A- and G-tailing reactions prevented detection of a terminal-nucleotide preference, which may be negligible. In contrast, in C- and T-tailing reactions, FAM69T was the least favorable substrate, followed by FAM69A. Comparison of reaction products using FAM69G and FAM70 as substrates showed that tailing of phosphorylated and non-phosphorylated ends do not differ considerably. However, the phosphate group at the opposite 5′ end slightly enhanced A- and G-tailing reactions and reduced C- and T-tailing reactions.

### Effect of DNA length on G-tailing reactions

**Figure 4:**
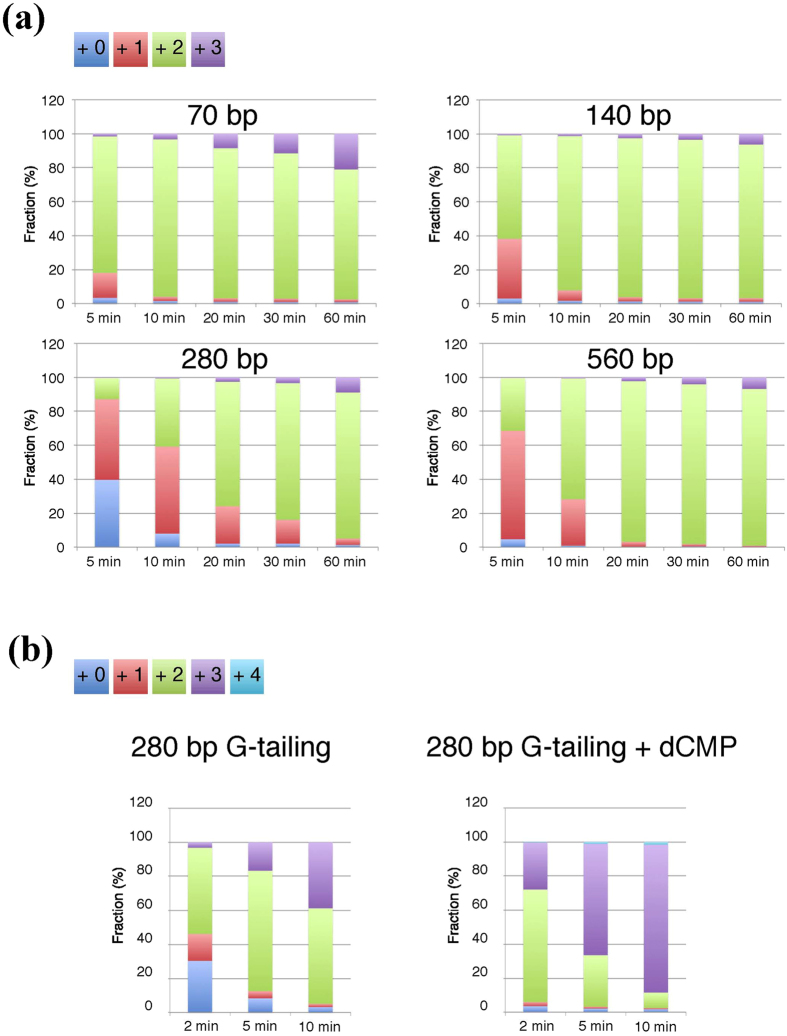
Tailing efficiencies of DNA molecules with different lengths that share the same last seven bases. (**a**) Tailing kinetics of four shorter molecules. Six FAM-labeled molecules with 70, 140, 280, 560, 840, and 1,120 bp sharing the same 7-bp 3′ sequence were prepared (see Supplementary Fig. S2) and 50 fmols of each (300 fmols in total) were mixed and incubated with MMLV-RT in the presence of dGTP. The reaction products were analyzed, and the size distribution of the tailing products of the four smaller molecules was determined. (**b**) dCMP stimulates tailing of the 280-bp DNA. In total, 100 fmols of the least efficient substrate DNA with 280 bp was subjected to G-tailing in the absence (left) or presence (right) of 2 mM dCMP. Results are shown as averages of triplicate experiments.

To investigate the effect of DNA length on the G-tailing reaction, FAM-labeled DNA molecules with 70, 140, 280, 560, 840, and 1,120 bp were prepared by PCR (Fig. S1b). In our attempt to exclude the effect of the terminal nucleotide sequence on G-tailing, the DNA substrates shared the same stretch of seven nucleotides at the tailing-target end. The six molecules mixed at equimolar ratios were subjected to G-tailing. Figure 4a shows the progression of the G-tailing reaction on the 70-, 140-, 280-, and 560-bp molecules (an increase in size was observed for the two longer fragments, but quantification was not possible due to poor peak resolution). We noted that longer fragments tended to acquire G-tails more slowly, and we investigated this issue in more detail (see bellow).

### G-tailing efficiency unaffected by DNA length

**Figure 5:**
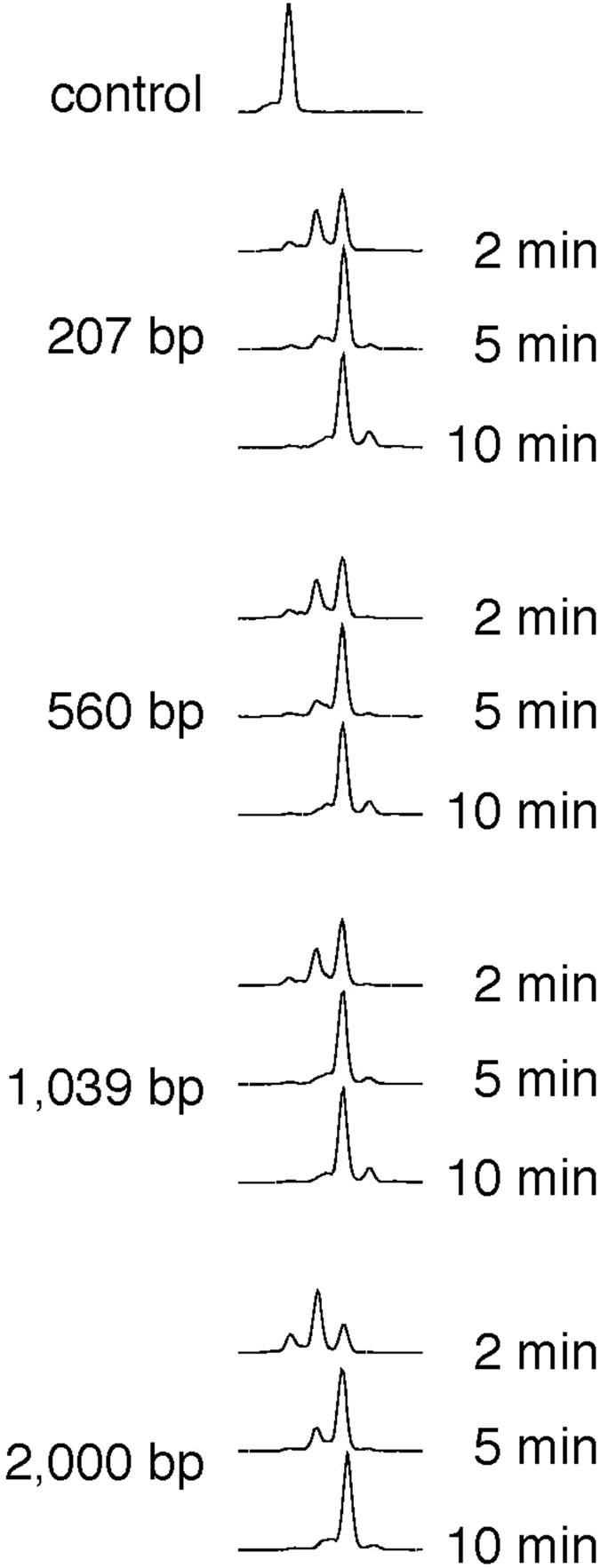
Tailing efficiencies of DNA molecules with different lengths that share the same 207-bases sequence. Four DNA molecules with the indicated sizes sharing the same 207-bp nucleotide sequence at the tailing target end were subjected to G-tailing for 2, 5, and 10 min in the absence of dNMP (see Supplementary Fig. S2 for experimental details). Reaction products were modified as described in Methods, and the product lengths were analyzed. Untreated control of the 207-bp molecule is shown at the top.

To investigate in more detail the effect of DNA length on tailing reactions, DNA molecules with 207, 560, 1,039, and 2,000 bp were assayed for end-tailing (Fig. 5 and Supplementary Fig. S2c). The four molecules contained a 207-bp identical sequence at the target end. After the G-tailing reaction, the DNA molecules were cleaved at the DraII site located 169 bp from the target end and ligated to a FAM-labeled adapter DNA with a complementary overhang, resulting in a fragment (205 bp plus tail) short enough to be analyzed by the sequencer. As shown in Fig. 5, the three shorter molecules exhibited indistinguishable kinetics, and the longest molecule exhibited a slightly slower kinetics. The observed reduction in kinetics was small (for comparison see Fig. 4a), indicating that DNA length does not affect tailing activity. In contrast, the total amount of DNA in a reaction affected the kinetics, which is consistent with our findings that uncut lambda DNA slightly inhibited tailing and that MMLV-RT bound to double-stranded DNA in electrophoresis mobility shift assay (data not shown).

### Negligible 3′ to 5′ exonuclease activity of MMLV-RT

The tailing reaction products of MMLV-RT might be the result of unbalanced activities of nucleotide addition and removal. To test whether MMLV-RT removes 3′ overhang nucleotides, G-tailed products were purified using a DNA purification column followed by ethanol precipitation to ensure the complete removal of dGTP used in the tailing reaction. G-tailed products were then incubated with MMLV-RT in the absence of dNTP and in the presence or absence of Mn^2+^. The G-tails remained unaffected after four h of incubation (data not shown), indicating that the 3′ to 5′ exonuclease activity of MMLV-RT is negligible.

### Ligation of adapter DNA to G-tailing reaction products

**Figure 6:**
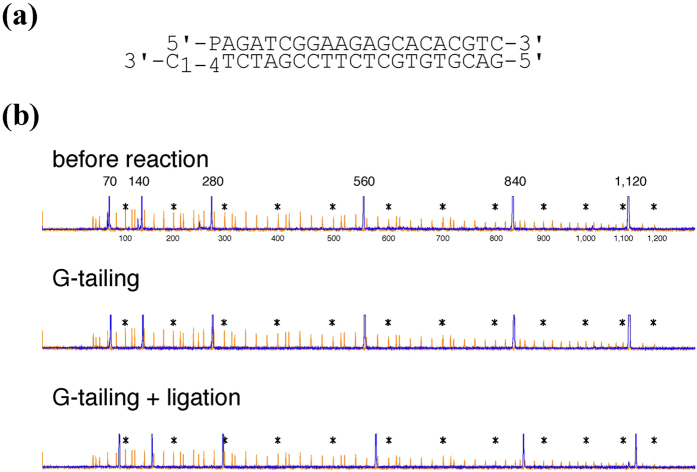
G-tailing products are efficiently ligated to adapter DNAs. (**a**) Adapter DNA used. The 5′ end of the top strand contains a phosphate group, and the 3′ end of the bottom strand contains C1 to C4 overhangs. (**b**) Electropherogram showing efficient adapter ligation. Mixture of the six FAM-labeled molecules with 70, 140, 280, 560, 840, and 1,120 bp were analyzed before (top) and after G-tailing reaction without dNMPs (middle), and after subsequent ligation with the adapters (bottom) shown in (**a**). Samples were mixed with the LIZ1200 size standard and analyzed using the sequencer. Blue and orange lines represent FAM and LIZ signals, respectively. LIZ peaks with sizes of 100 × n (where n is a number from 1 to 12) are marked with asterisks.

To test whether G-tailing products could be ligated to adapter DNA containing a C-overhang, a mixture of six different molecules were G-tailed for 30 min, purified, and subjected to ligation with a mixture of four kinds of adapters, each containing a C-tail with 1 to 4 nucleotides. Because ligation increases length by more than 20 nucleotides, the ligated products could easily be distinguished from the non ligated substrate (Fig. 6). The adapter was ligated to 91% ± 1% of the 1,120-bp molecules and to more than 97% of each of the five shorter molecules, indicating that G-tailing could be used for efficient ligation of adapter DNAs.

## Discussion

In this study, efficient A-, C-, G- and T-tailing activities that append 3′ overhangs to double-stranded DNA ends by MMLV-RT^11^,^16^ were demonstrated. The tailing activity is strong enough to change most of the substrate DNA into tailed products. In terms of tailed product ratios, tail lengths, and the versatility to append any nucleotide, MMLV-RT is superior to known DNA polymerases that catalyze template-independent nucleotide addition^6^,^7^,^8^,^9^. The exo^−^ mutant of the Klenow fragment^7^ efficiently appends +1 A and +1 G tails but not +1 C or +1 T tails, and Taq DNA polymerase appends +1 A tails^6^ but almost half the substrate remained untailed even after prolonged incubation (data not shown). Although the physiological role of these properties is beyond the scope of this study, MMLV-RT may be used in additional biotechnological applications together with other fundamental DNA manipulation techniques.

The mechanisms by which incorporation of dNTP is enhanced by the addition of complementary dNMP, as observed for the exo^−^ mutant of the Klenow fragment^7^, remain elusive. Our results show that the first and second nucleotides are added faster than subsequent nucleotides, suggesting that addition of a dNTP to the 3′ end by MMLV-RT relies on its interaction with the 5′ end of the opposite strand. This is consistent with the observation that the 5′ phosphate affects tailing reactions. As the 3′ tail is extended and the 5′ end becomes recessed relative to the 3′ end, this interaction is lost. In this context, a dNMP may interact with MMLV-RT at the site that would otherwise interact with the 5′ end of the opposite strand, thereby stabilizing the incoming dNTP by transient Watson-Crick pairing and thus enhancing the tail-extension reaction. In contrast, dATP incorporation was not enhanced by any one of the four dNMPs. The preferred template-independent incorporation of dATP by DNA polymerases might be due to the strong intrahelical base-stacking nature of dATP^8^. The strong base-stacking of dATP might also account for the observation that dTMP does not enhance the incorporation of dATP.

MMLV-RT showed steady but slow tailing activity on blunt DNA ends with A or T at its 3′ terminus. In addition, target DNA length per se, that is, the distance from the other DNA end, does not affect tailing speed (Fig. 5). The results presented in Fig. 4 raised the possibility that long fragments acquire 3′ tails less efficiently. We first postulated a mechanism, in which MMLV-RT enters from one DNA end and traverses along the molecule to reach the opposite end and then appends a nucleotide. This mechanism predicts that two MMLV-RT molecules coming from the both two ends would collide, resulting in less efficient appendage, which is reminiscent of the collision between the RNA polymerase and DNA replication apparatuses^17^ and between two RNA polymerase molecules^18^. If this is the case, biotechnological applications of MMLV-RT would be limited. To investigate the effects of DNA length on tailing efficiency, we developed an experimental scheme (Fig. S2) and showed that target DNA length is not a determinant of the tailing efficiency. Although these determinants are still unknown, molecules with different sequences and lengths but with the same last seven nucleotides acquired a tail at different speeds. These results suggest that tailing reaction efficiency might be influenced by terminal nucleotide composition, and properties of the end-proximal region of DNA such as GC-content, DNA conformation, and/or presence of unknown DNA sequences that direct the MMLV-RT to DNA ends.

Although some DNA molecules acquired 3′ tails slowly, we showed that dAMP, dCMP, and dGMP enhance T-, G-, and C-tailing by MMLV-RT, respectively. For instance, the worst substrate among the six molecules tested, acquired, in the presence of dCMP, a G-tail at a speed exceeding that of the best substrate (compare Fig. 4b and Fig. S1A). The presence of dNMP thus ensures the addition of at least one overhang for subsequent reactions at a speed sufficient for biotechnological applications.

The distribution of overhang length varies depending on incubation period, the 3′ end nucleotide, DNA sequence at the end-proximal region, the kind of dNTPs used, presence of complementary dNMP, and amount of MMLV-RT used. However, it is expected that any DNA molecule at 10 nM or less can be tailed with at least one nucleotide by incubation with 50 U of MMLV-RT for 10 min at 30 °C in the presence of 4 mM Mn^2+^ and 10 mM complementary dMNP. The maximum tail length observed after prolonged incubation was 6, 7, 4, and 4 for A-, C-, G-, and T-tails, respectively. Such a small tail length is favorable to adapter ligation, and a mixture of adapter DNAs with different complementary-overhang lengths can be efficiently ligated to the tailed ends. It may also be possible to utilize MMLV-RT to add complementary overhangs to double-stranded adapter DNA with one 3′ end blocked to enable tailing of the other 3′ end only.

In this study, we used a capillary DNA sequencer to detect size changes in FAM-labeled DNA strands. Use of a sequencer is advantageous over traditional denatured polyacrylamide gel electrophoresis in that it can quickly separate DNAs with a wide range of lengths (up to approximately 700 nucleotides) at a one-base resolution. In our experimental system, using a sequencer with 16 capillaries allowed processing of 96 samples for DNA size increase within three h. Although the use of a capillary DNA sequencer is common in genotyping^19^,^20^ and footprinting analyses^21^, it is apparently still rare for DNA length analyses that use DNA-modifying enzymes. Suitable computational tools may have not yet to be developed for this purpose. To facilitate such analyses, we developed a computational tool called TraceViewer, which reads trace data in ABIF format and calibrates runs using the mobility of two user-selected internal control fragments of different sizes that flank the peaks of interest. By using this software, changes in DNA length ranging from 10 to 700 nucleotides are readily detectable at single nucleotide resolution. The software was used for precise determination of the nick site of plasmid NAH7 in its conjugative transfer^22^, and also for DNase I footprinting (data not shown). TraceViewer exports the areas of selected peaks, and adjusts the apparent peak heights so that the sum of selected peak areas is apparently even across different samples. TraceViewer also exports vector graphics for presentations.

The efficient addition of nucleotides to blunt-ended DNA by MMLV-RT to generate 3′ overhangs may be the basis of a breakthrough technology that, combined with the use of unique molecular identifiers, may enable the exhaustive analysis of a given DNA pool. In addition, the use of strong tailing activity reduces the amount of DNA needed for analysis, which will be useful for single-cell or ancient DNA analyzes. Although both DNA ends may be subjected to tailing and currently no means to append different tails to both ends of naturally occurring DNA are available, the enzymatic property of MMLV-RT described in this work shows the potential for many biotechnological applications.

## Additional Information

**How to cite this article**: Ohtsubo, Y. *et al*. Efficient N-tailing of blunt DNA ends by Moloney murine leukemia virus reverse transcriptase. *Sci. Rep.* **7**, 41769; doi: 10.1038/srep41769 (2017).

**Publisher's note:** Springer Nature remains neutral with regard to jurisdictional claims in published maps and institutional affiliations.

## Supplementary Material

Supplementary Data
