# Supplementary material for: Efficient N-tailing of blunt DNA ends by Moloney murine leukemia virus reverse transcriptase
Source: Sci Rep. 2017 Feb 2;7:41769. doi: 10.1038/srep41769 (PMC5288710; doi:10.1038/srep41769)
Supplement: Supplementary Data [file srep41769-s1.pdf]

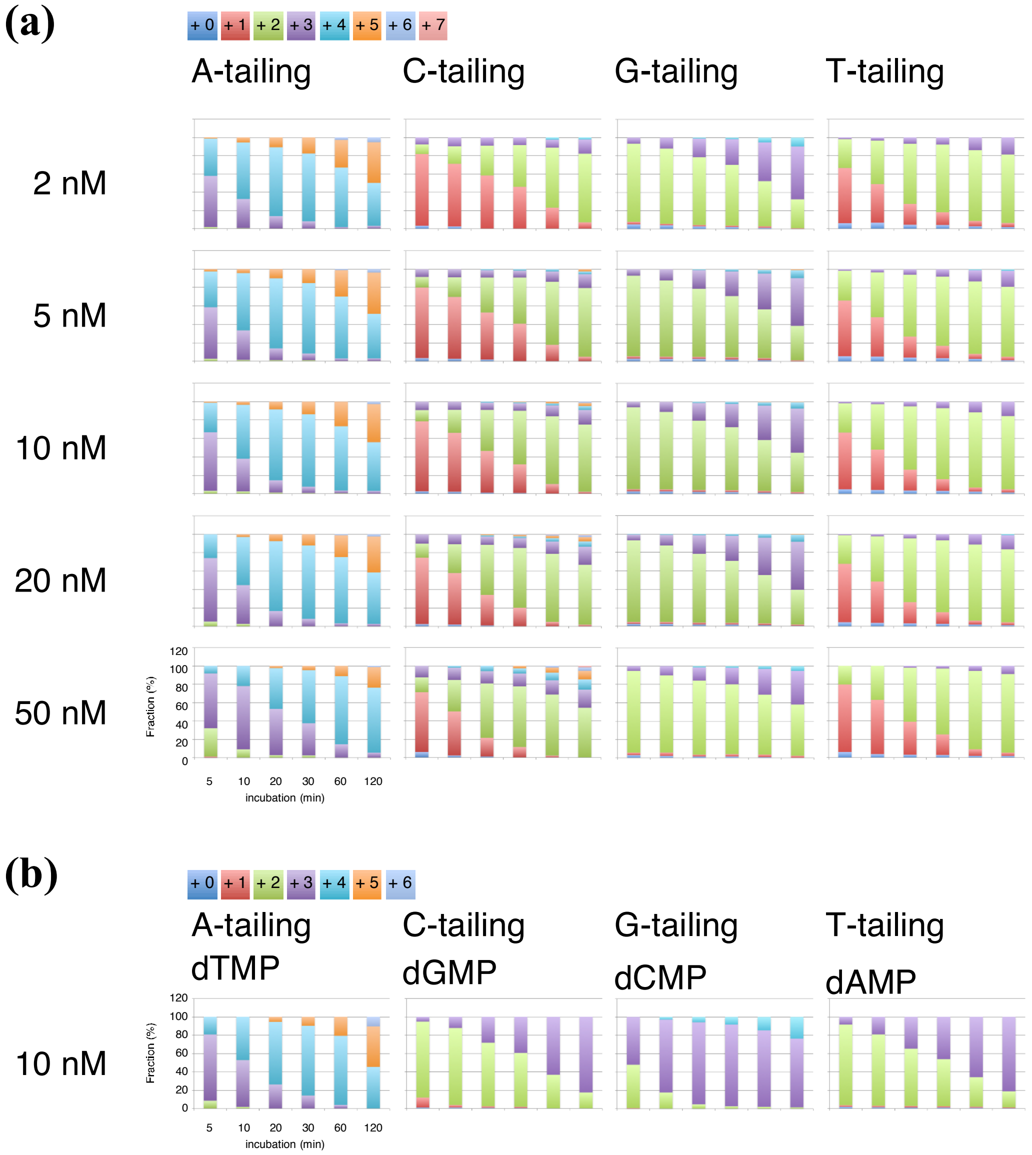

Supplementary Fig. 1. Kinetics of tailing reactions. (a) Tailing of different concentrations of FAM70. FAM70 DNA from 2 nM to 50 nM was subjected to A-, C-, G-, and T-tailing reactions in the absence of dNMPs. **(b) dNMP assisted tailing of FAM70 DNA.** Ten nM FAM70 DNA was subjected to A-, C-, G-, and T-tailing reactions in the presence of dTMP, dGMP, dCMP, and dAMP, respectively. For simplicity, only the bottom-left graph in each panel is labeled. Results are shown as averages of triplicate experiments.

Supplementary Fig.S1

Efficient N-tailing of blunt DNA ends by Moloney murine leukemia virus reverse transcriptase

Yoshiyuki Ohtsubo, Yuji Nagata, and Masataka Tsuda

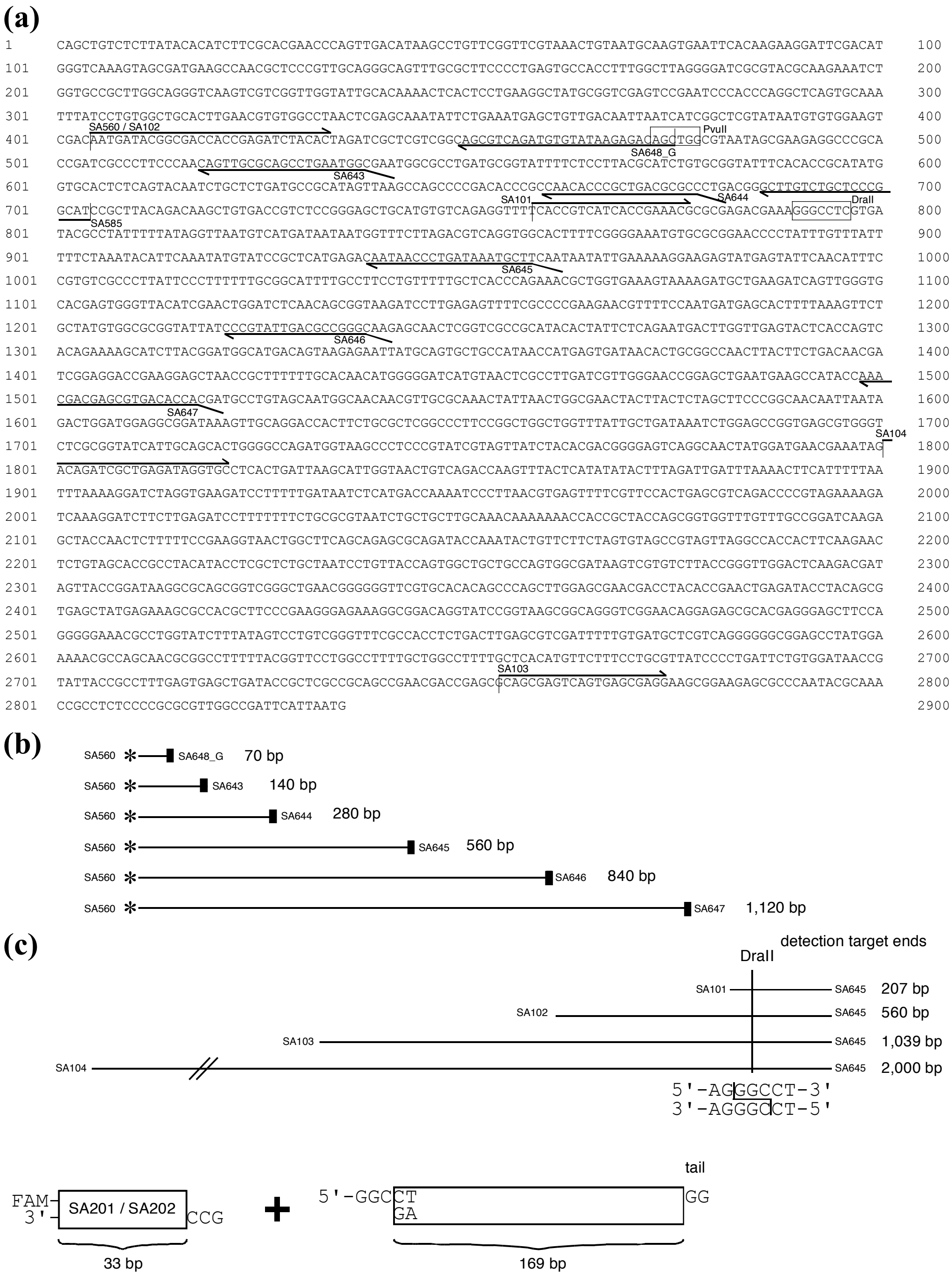

Supplementary Fig. 2. DNA fragments used in this study. (a) Nucleotide sequence of plasmid pGiTp used as a template for PCR amplifications. The primer-annealing sites are indicated by arrows above and below the sequence for forward-oriented and reverse-oriented primers, respectively. The primer tails correspond to the sequence 5'-CTGTCTCT-3', which were included to make the last seven bases of the PCR products identical (indicated by black boxes in panel b). **(b) The six FAM-labeled molecules used in this study.** Each molecule is labeled with the names of the two primers used. Asterisks represent FAM fluorophores and black boxes represent an identical DNA tract of seven bases. **(c) Four DNA molecules used to investigate the effect of length on tailing efficiency.** Any larger molecule contains the sequence of the shorter molecule, and all molecules contain a DraII site located 169 bp from the right end. After DraII digestion, 5' FAM-labeled double-stranded adapters with an overhang of 5'-GCC-3' were ligated to the DraII-generated 169-bp fragment with an overhang of 5'-GGC-3' (shown at the bottom). Due to the asymmetrical overhangs generated by DraII, the adapter DNA does not ligate to the remaining fragments. Two Gs indicate the location of the tail.

Supplementary Fig.S2

Efficient N-tailing of blunt DNA ends by Moloney murine leukemia virus reverse transcriptase

Yoshiyuki Ohtsubo, Yuji Nagata, and Masataka Tsuda

Supplementary Table S1

name	sequence 5' ->3'	note
SA101	TCACCGTCATCACCGAAAC	To amplify 207-bp fragment with SA645
SA102	AATGATACGGCGACCACCGAGATCTACAC	To amplify 560-bp fragment with SA645
SA103	GCAGCGAGTCAGTGAGCGAG	To amplify 1,039-bp fragment with SA645
SA104	GACAGATCGCTGAGATAGGTGC	To amplify 2,000-bp fragment with SA645
SA201	GCCCTGTCTCTTATACACATCTGACGCTGCCGACGA	Anneals with SA202 to make adaptor DNA with 5' extension of GCC
SA202	6FAM-TCGTCGGCAGCGTCAGATGTGTATAAGAGACAG	Anneals with SA201
SA560	6FAM-AATGATACGGCGACCACCGAGATCTACAC	To amplify fragments; used with SA643-647 and SA648-SA651
SA576_C4	GACGTGTGCTCTTCCGATCTCCCC	Anneals with SA582, four C-overhang
SA577_C3	GACGTGTGCTCTTCCGATCTCCC	Anneals with SA582, three C-overhang
SA578_C2	GACGTGTGCTCTTCCGATCTCC	Anneals with SA582, two C-overhang
SA579_C1	GACGTGTGCTCTTCCGATCTC	Anneals with SA582, one C-overhang
SA582	P-AGATCGGAAGAGCACACGTC	Phosphorylated at 5' end; Anneals with SA576-SA579
SA585	ATGCCGGGAGCAGACAAGCC	To amplify 300-bp fragment with SA560
SA643	CTGTCTCCCATTCAGGCTGCGCAACTG	To amplify 140-bp fragment with SA560
SA644	CTGTCTCGCGCGTCAGCGGGTGTTGG	To amplify 280-bp fragment with SA560
SA645	CTGTCTCAGCATTTATCAGGGTTATTG	To amplify 560-bp fragment with SA560; also used with SA101-SA104
SA646	CTGTCTCCCCGGCGTCAATACGGG	To amplify 840-bp fragment with SA560
SA647	CTGTCTCTGGTGTCACGCTCGTCGTTTG	To amplify 1120-bp fragment
SA648_G	CTGTCTCTTATACACATCTGACGCTG	To ampfify FAM69G with SA560
SA649_C	GTGTCTCTTATACACATCTGACGCTG	To ampfify FAM69C with SA560
SA650_T	ATGTCTCTTATACACATCTGACGCTG	To ampfify FAM69T with SA560
SA651_A	TTGTCTCTTATACACATCTGACGCTG	To ampfify FAM69A with SA560

Efficient N-tailing of blunt DNA ends by Moloney murine leukemia virus reverse transcriptase

Yoshiyuki Ohtsubo, Yuji Nagata, and Masataka Tsuda
